# Supplementary material for: Quantitative single-cell interactomes in normal and virus-infected mouse lungs
Source: Dis Model Mech. 2020 Jun 26;13(6):dmm044404. doi: 10.1242/dmm.044404 (PMC7328136; doi:10.1242/dmm.044404)
Supplement: Supplementary information [file dmm-13-044404-s1.pdf]

SUPPLEMENTAL INFORMATION

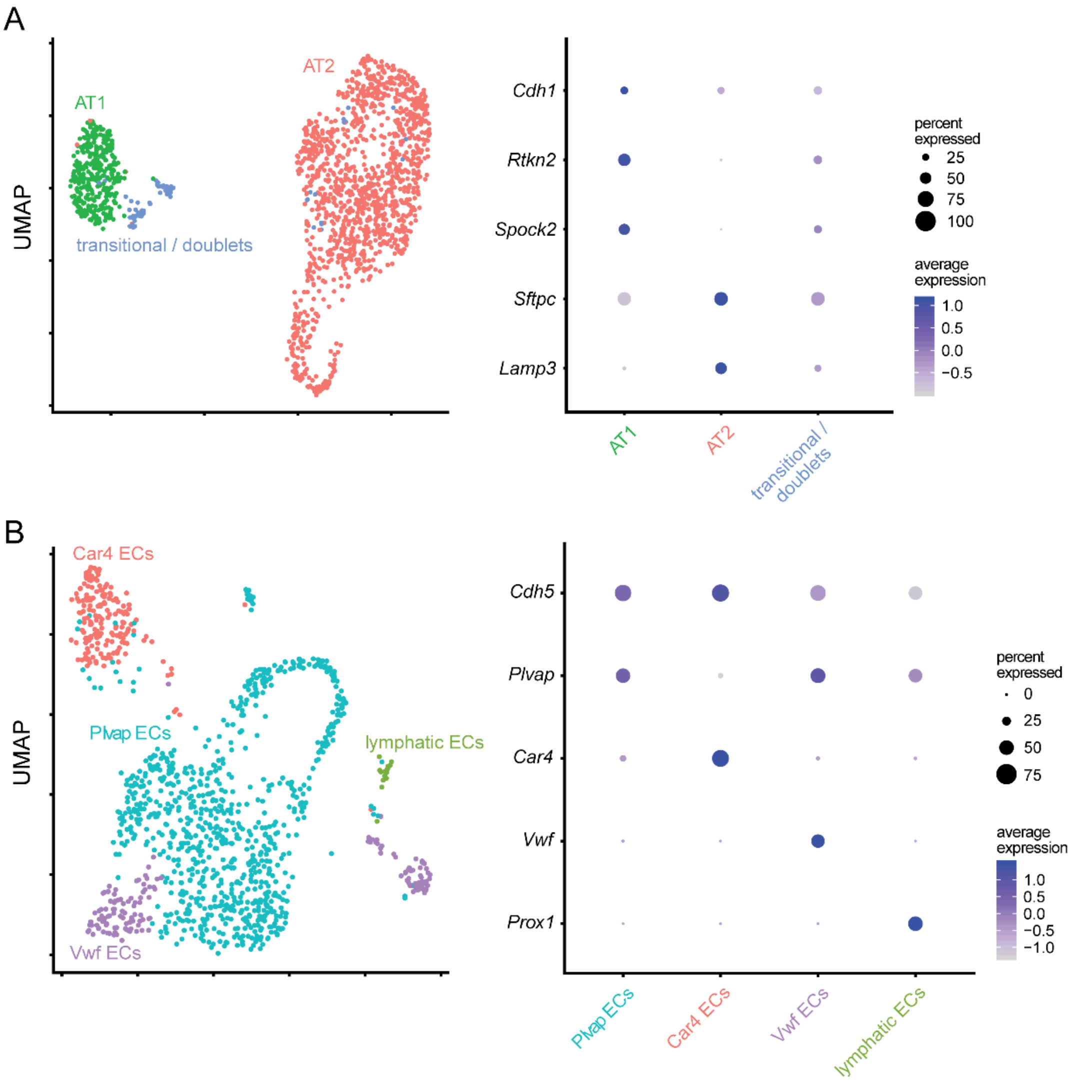

Fig. S1. Control P7 lung scRNA-seq data used in single-cell versus bulk RNA-seq analyses. (A) Uniform Manifold Approximation and Projections (UMAP) plots (left) of scRNA-seq of P7 lung epithelial cells, as identified by their marker genes in a dot plot (right). Transitional/doublets express both AT1 and AT2 marker genes and are not included in subsequent analysis. (B) UMAP plots (left) of scRNA-seq of P7 lung endothelial cell populations, as identified by their marker genes in a dot plot (right).

Supplemental Tables

Table S1. Comparison of bulk RNA-seq and scRNA-seq gene expression in AT1, AT2, and endothelial cell populations.

Click here to Download Table S1

Table S2. Average ligand and receptor gene expression for the 18 cell types of control (worksheet 1) and Sendai virus-infected (worksheet 2) lungs.

Click here to Download Table S2

Table S3. Ligand-receptor interaction scores in each cell type pair in control (worksheet 1) lung and Sendai virus-infected (worksheet 2) lungs.

Click here to Download Table S3

Table S4. Binary indicators of outlying ligand-receptor interactions in each cell type pair in control (worksheet 1) and Sendai virus-infected (worksheet 2) lungs. 1 indicates outlying interaction.

Click here to Download Table S4

Table S5. Differences in ligand-receptor interaction in each cell type pair between control and infected lungs (worksheet 1) and their binary indicators (worksheet 2; 1, increased; -1, decreased; 0, no change).

Click here to Download Table S5

Table S6. GO pathway scores of each cell type in control (worksheet 1) lung and Sendai virus-infected (worksheet 2) lungs.

Click here to Download Table S6

Table S7. Binary GO pathway indicators of each cell type in control (worksheet 1) lung and Sendai virus-infected (worksheet 2) lungs. 1 indicates outlying pathways.

Click here to Download Table S7

Table S8. Differences in GO pathway scores in each cell type between control and infected lungs (worksheet 1) and their binary indicators (worksheet 2; 1, increased; -1, decreased; 0, no change).

Click here to Download Table S8

Table S9. Integration of outlying ligand-receptor interactions and GO pathways in each cell type (individual worksheets) in the control lung.

Click here to Download Table S9

Table S10. Integration of outlying differences in ligand-receptor interactions and GO pathways in each cell type (individual worksheets) between control and Sendai virus-infected lungs.

Click here to Download Table S10

Supplementary Data. R code for figures and supplemental tables.

Click here to Download Supplementary Data
